# The Relationship Between Static Characteristics of Physicians and Patient Consultation Volume in Internet Hospitals: Quantitative Analysis

**DOI:** 10.2196/56687

**Published:** 2024-06-17

**Authors:** Ye Wang, Changjing Shi, Xinyun Wang, Hua Meng, Junqiang Chen

**Affiliations:** 1 Internet Hospital Operation Department The First Affiliated Hospital of Guangxi Medical University Nanning China; 2 Information Center The First Affiliated Hospital of Guangxi Medical University Nanning China; 3 Department of Gastrointestinal Gland Surgery The First Affiliated Hospital of Guangxi Medical University Nanning China

**Keywords:** static characteristics of physicians, internet hospitals, telemedicine, statistical analysis, online consultation, web-based consultation, teleconsultation, physician, patient

## Abstract

**Background:**

Internet medical treatment, also known as telemedicine, represents a paradigm shift in health care delivery. This contactless model allows patients to seek medical advice remotely, often before they physically visit a doctor’s clinic. Herein, physicians are in a relatively passive position, as patients browse and choose their health care providers. Although a wealth of experience is undoubtedly a draw for many patients, it remains unclear which specific facets of a doctor’s credentials and accomplishments patients prioritize during their selection process.

**Objective:**

Our primary aim is to delve deeper into the correlation between physicians’ static characteristics—such as their qualifications, experiences, and profiles on the internet—and the number of patient visits they receive. We seek to achieve this by analyzing comprehensive internet hospital data from public hospitals. Furthermore, we aim to offer insights into how doctors can present themselves more effectively on web-based platforms, thereby attracting more patients and improving overall patient satisfaction.

**Methods:**

We retrospectively gathered web-based diagnosis and treatment data from the First Affiliated Hospital of Guangxi Medical University in 2023. These data underwent rigorous analysis, encompassing basic descriptive statistics, correlation analyses between key factors in doctors’ internet-based introductions, and the number of patient consultation visits. Additionally, we conducted subgroup analyses to ascertain the independence of these vital factors. To further distill the essence from these data, we used nonnegative matrix factorization to identify crucial demographic characteristics that significantly impact patient choice.

**Results:**

The statistical results suggested that there were significant differences in the distribution of consultation volume (*P*<.001), and the correlation analysis results suggested that there was a strong correlation between the two groups of data (ρ=0.93; *P*<.001). There was a correlation between the richness of a profile and popularity (*P*<.001). Patients were more interested in physicians with advanced titles, doctoral degrees, social activities, and scientific achievements (*P*<.001) as well as other institutional visit experiences (*P*=.003). More prosperous social activities, scientific achievements, experiences of other institutional visits, and awards were more common among people with advanced professional titles. Doctoral degrees remained attractive to patients when data were limited to senior physicians (*P*<.001). Patients trusted the medical staff with advanced titles, social activities, scientific achievements, and doctoral degrees (*P*<.001).

**Conclusions:**

Patient preferences for choosing a health care provider differed significantly between free and paid consultations. Notably, patients tended to trust doctors with advanced professional titles more and were more likely to seek out those with doctoral qualifications over other professional ranks. Additionally, physicians who actively participated in social events and scientific endeavors often had an advantage in attracting new patients. Given these insights, doctors who invest in enhancing their personal and professional experiences within these domains are likely to see increased popularity and patient satisfaction.

## Introduction

**Figure 1:**
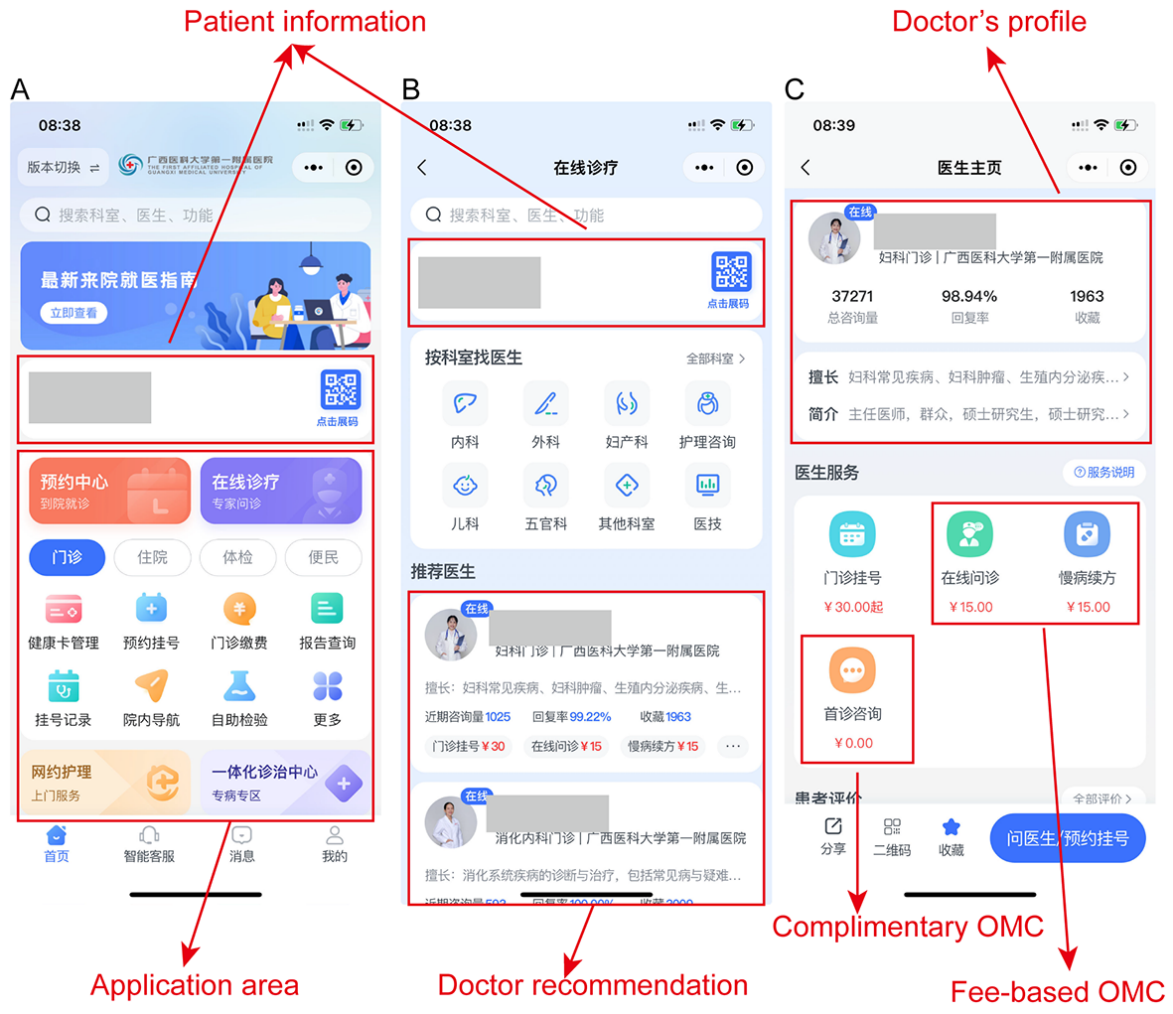
Screenshot of an internet hospital platform. OMC: online medical consultation.

The online medical consultation (OMC) is a new and rapidly developing telemedicine model. Patients can spend little time traveling and waiting in line. They can ask physicians health-related questions and information about disease treatments via the internet. Recent studies have shown that remote monitoring systems demonstrate the potential to enhance patient self-management and health care efficiency [1]. For instance, the iGetBetter system strengthens the connection between patients with heart failure and their medical teams [2], while the PIMPmy Hospital app is expected to optimize laboratory result access processes in pediatric emergency rooms [3]. In their study, McCrabb et al [4] conducted a web-based cross-sectional survey of patients with orthopedic trauma from 2 public hospitals in New South Wales, Australia, and found that more than half of the respondents expressed interest in using the internet to promote their health. In 2018, China began actively developing the “Internet + medical and health” system, providing web-based services, such as medical appointments, chronic disease follow-up, and telemedicine. Internet hospitals are almost a unique OMC model in China. As of June 2023, there have been more than 3000 internet hospitals in China carrying out internet diagnosis and treatment services for more than 25.9 million people, and the scale of internet hospitals has increased by about 1300 compared with the end of 2021 [5]. Yu et al [6] confirmed the feasibility of implementing shared decision-making between doctors and patients in the diagnosis and treatment services of internet hospitals by analyzing the characteristics and medical needs of patients at the Tianjin Medical University General Hospital Internet Hospital, providing a new approach to enhancing patients’ medical experience. The organization can rely on the medical institution entity to establish an internet hospital to provide patients with safe and appropriate medical services via the internet [7]. Taking the First Affiliated Hospital of Guangxi Medical University as an example， the OMC service functions include a doctor search box, a patient information bar, medical staff team classification, a recommended doctor information column, recommended doctor details, an artificial intelligence preconsultation module, a doctor-patient communication box, an electronic medical record display module, a prescription information module, medicine purchase method, and an order payment module (Figure 1). Therefore, based on the entity hospital, the internet hospital is only the beginning of the disease management of patients [8,9].

The “personal advertisement” of physicians on the internet hospital is the first-hand objective information that patients can consult, and the “quality of advertisement” determines whether physicians can have new customers. Qiu et al’s [10] research found that factors like doctors’ medical titles, the reputation of the department where they work, the number of gifts received, and the number of patients registered after seeing a doctor have significant positive effects on choosing a doctor. According to Chen et al’s [11] research, physicians’ titles and city level weakly impact the number of physicians’ consultations. In contrast, the number of patient visits completed, the number of gifts received, and the number of articles published positively impact the number of physicians’ teleconsultations. Based on the analysis of eye tracking and questionnaire data, Shan et al [12] finally concluded that favorability, consultation frequency, professional title, hospital, satisfaction, profile photo, and cost are critical influencing factors for patients to choose physicians. Data from China’s Good Doctor suggest that the more types of services physicians open and the more articles they publish as well as the use of personal avatars will promote the patient’s choice of medical consultation services [13]. Unlike other published literature on OMCs, our internet hospital is supported by entity hospitals. When choosing a remote doctor, patients may consider visiting the hospital in person, which is helpful to the hospital’s economic benefits and disease management. In addition, patients can transition from web-based consultations in internet hospitals to in-person hospitalization. Our previous research results suggest that from 2020 to 2022, the consulting volume of senior professional titles was much higher than that of intermediate and low professional titles [14]. At present, most of the research on factors influencing patients’ medical choices based on internet hospitals discusses the quality of web-based medical service [15,16]; examples include perception factors [17,18] and service quality assessment [19]. Few studies have reported on factors influencing patients’ choices in internet hospitals inside public hospitals. The third-party web-based hosting platforms, such as “Good Doctor,” “Chunyu Doctor,” “Ping a Good Doctor,” and “Wedoctor,” have more physicians and richer sources of patients [20]. The services of internet hospitals in public hospitals are subject to geographical restrictions, and the choice of internet hospitals for treatment is influenced by both patient factors [21,22] and public hospital factors [23]. Each characteristic factor in the personal advertisement is derived from the doctor’s career, reflecting the doctor’s comprehensive ability, reputation, and integrity. Therefore, we urgently need to learn which factors are more attractive to patients, influencing their trust.

**Figure 2:**
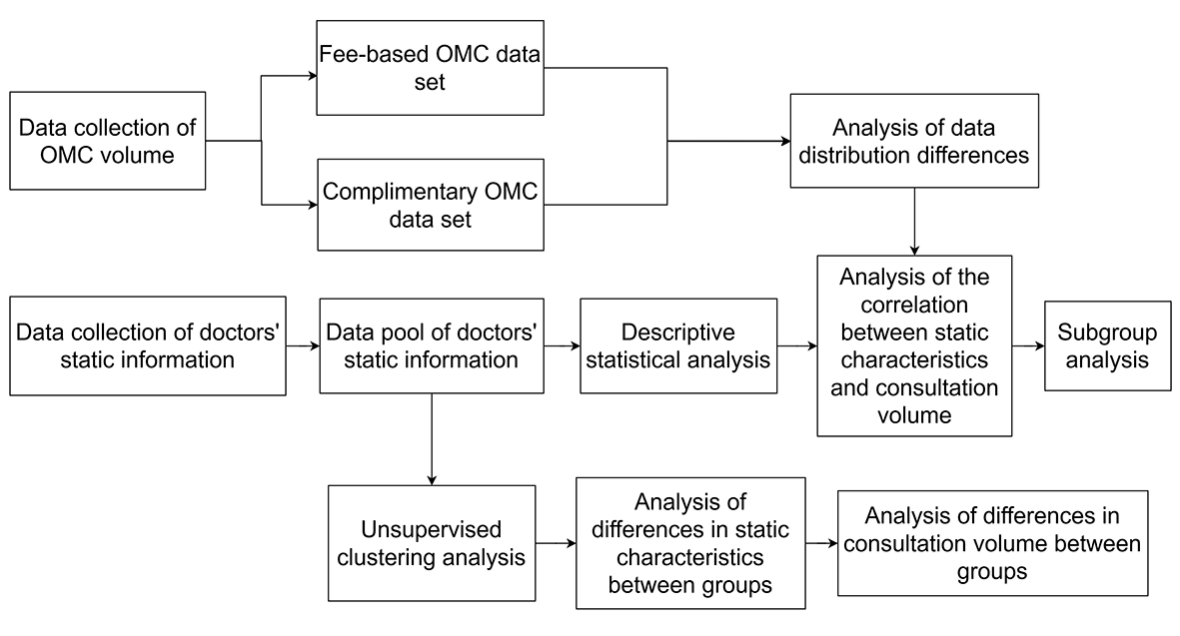
The flow chart of research design. OMC: online medical consultation.

Overall, this study aimed to establish a data pool of doctors’ static information based on the personal information displayed by doctors in internet hospitals. By combining these data with web-based consultation volume data, the study further analyzed the relationship between static information characteristics and web-based consultation volume. Subsequently, unsupervised clustering analysis was used to categorize doctors into different groups. Descriptive statistical analysis was then used to explore the static information characteristics of each group and compare the differences in web-based consultation volumes among groups. Based on the above analysis results, the study ultimately identified the key factors influencing patients’ medical treatment behaviors (Figure 2).

## Methods

### Data Collection

The OMC data of the First Affiliated Hospital of Guangxi Medical University in 2023 were retrospectively collected and sorted out, including each doctor’s patient diagnosis and treatment visits, patient consultation visits, personal introduction, and business skills introduction. The doctor’s personal introduction and professional skill introduction were edited and uploaded by the doctor and published after the hospital’s review. The parameters were defined as follows: the title type is divided into specialists (ie, chief physicians with more than 5 years of service, chief physician, deputy chief physician, attending physician, and resident physician). Among them, specialists, chief physicians, and deputy chief physicians were defined as advanced professional titles, attending physicians were defined as intermediate professional titles, and resident physicians were defined as junior professional titles. People who participated in a political party were recorded as “yes.” Degrees were classified as doctoral degree, master’s degree, and “undescribed” if no relevant information was available. Postdoctoral research experience, postgraduate supervisor experience, administrative positions in hospitals, participation in social organization activities, published papers and scientific research, records of journal editorial board membership, other institutional visit experiences, and awards were all recorded as “yes,” if applicable. If there was no relevant information, it was recorded as “undescribed.”

Inclusion criteria involved doctors participating in OMC services throughout the year 2023; exclusion criteria involved doctors who had closed OMC services for more than 30 days.

### Ethical Considerations

This study was reviewed and approved by the Ethics Committee of the First Affiliated Hospital of Guangxi Medical University, ensuring strict protection of the privacy and confidentiality of human subjects (KY-0-34). We have anonymized or deidentified the research data. In the research results, we ensured that no identifiable individual images were included, fully respecting and protecting the rights and interests of participants.

### Statistical Method and Study Design

A nonparametric (Mann-Whitney test or Wilcoxon paired 2-tailed *t* test) or parametric (unpaired or paired 2-tailed *t* test) statistical test was used to compare differences between unpaired groups (Mann-Whitney test or unpaired 2-tailed *t* test) or paired groups (Wilcoxon paired test or paired 2-tailed *t* test). Spearman ρ correlation was used to assess associations. Descriptive statistics was performed using Microsoft Excel. Nonnegative matrix factorization (NMF) was performed by the “nmf” package in R, and the density plot was generated using R software (version 4.2.2; R Core Team) with package “ggplot2” (version 3.4.0). Statistical analysis was performed using SPSS (version 25, IBM SPSS) and GraphPad Prism (version 9.0; GraphPad Software Inc). *P*<.05, *P*<.001, and *P*<.0001 were considered statistically significant. The flow chart of the study design is shown in Figure 2.

## Results

### Baseline Data Information

**Figure 3:**
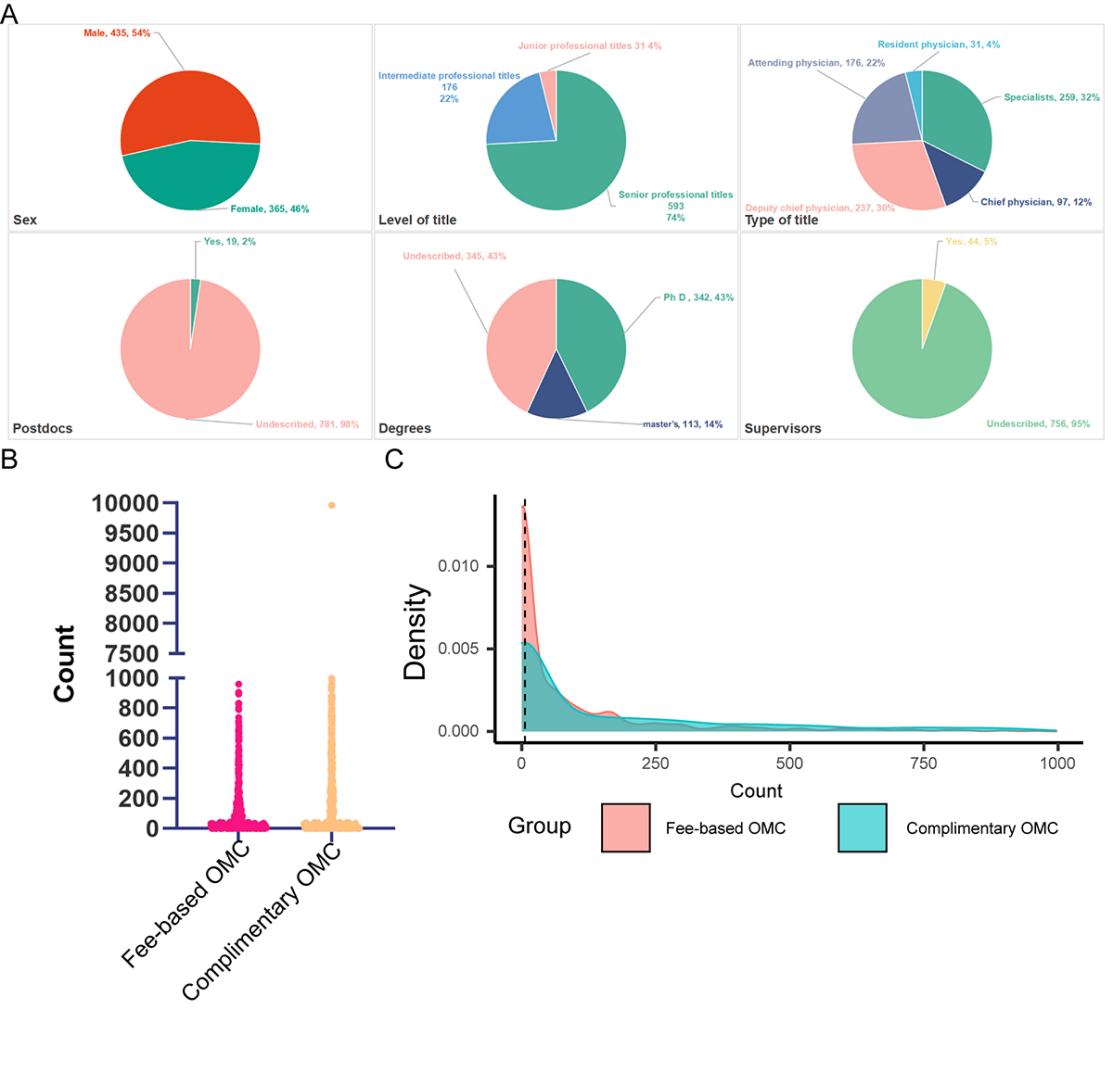
Internet hospital doctor static information data. (A) 13 vector factor distribution ratio results (some of the content is shown in Multimedia Appendix 1); (B and C) The data distribution of online medical consultation (OMC) volume. In 2023, 800 participants from 57 in-person clinics were included in the follow-up study, and only 74 physicians had more than 1000 consultations; most had fewer than 200 visits.

In 2023, a total of 975 physicians provided patient care services, of which 175 did not offer televisis. The remaining 800 participants from 57 in-person clinics were included in the follow-up study. The data were sorted by level of title, type of title, political party members, degrees, postdoctoral research experience, supervisory roles, administrative positions, social activities, scientific achievements, journal editorial board membership, other institutional visits, awards, and sex. The results suggested that there were 593 senior professional titles, 176 intermediate professional titles, and 31 junior professional titles. Additionally, there were 259 specialists, 97 chief physicians, 237 deputy chief physicians, 176 attending physicians, 31 resident physicians, and 144 party members. Furthermore, 342 held PhD degrees, 113 held master’s degrees, 19 had postdoctoral experience, 44 were supervisors, 123 had administrative positions, 407 participated in social activities, 329 had scientific achievements, 75 were journal editorial board members, 134 had other institutional visit experiences, and 186 had received awards. In terms of gender, 365 were female, and 435 were male. Among them, 26 specialists, 6 chief physicians, 23 deputy chief physicians, 30 attending physicians, and 1 resident physician provided title information. Detailed information is shown in Figure 3A and Figure S1 in Multimedia Appendix 1. The fee-based OMC volume in 2023 was 275,641 people counts, and the data did not conform to the normal distribution. The median was 29 (IQR 0-371.3), and the mean was 344.6 (SD 758.2). In 2023, there were 85,658 complimentary OMC counts. The data did not conform to the normal distribution (median 6, IQR 0-102; mean 107.1, SD 249). The data distribution of OMC volumes is shown in Figure 3B and 3C. The results indicated that the data were significantly different (*P*<.001). Spearman correlation analysis indicated that the correlation coefficient of the two groups was 0.93 (*P*<.001).

### Correlation Analysis

The number of words in the personal introduction was significantly different from the distribution of complimentary OMCs (*P*<.001) and fee-based OMCs data (*P*<.001), with Spearman correlation coefficients of 0.26 and 0.24, respectively. There was no significant difference in the number of skill introduction words and fee-based OMC data distribution (*P*=.85), but there was a significant difference from the complimentary OMC data distribution (*P*<.001). Spearman correlation coefficients were 0.36 and 0.33, respectively. The data are presented in Table S1 in Multimedia Appendix 1. The 2-tailed Wilcoxon test results indicated a significant difference in the number of fee-based OMC visits between senior and junior professional titles (*P*=.02), while there was no such difference in complimentary OMC data (*P*=.19). There was no difference in complimentary OMCs and fee-based OMCs between intermediate (*P*=.43) and junior professional titles (*P*=.10). There was a significant difference in the number of fee-based OMC visits among the chief physicians, deputy chief physicians (*P*=.01), and resident physicians (*P*<.001), while there was no such difference in the number of complimentary OMC visits (deputy chief physician: *P*=.14; resident physician: *P*=.08). There was a significant difference between the PhD and other degrees for complimentary OMC (*P*<.001) and fee-based OMC (*P*<.001) visits. Similar results were found for social activities (complimentary OMC and fee-based OMC visits: *P*<.001), scientific achievements (complimentary OMC and fee-based OMC visits: *P*<.001), visits to other institutions (complimentary OMC and fee-based OMC visits: *P*<.001), and awards (complimentary OMC visits: *P*<.001; fee-based OMC visits: *P*=.04). Detailed data are shown in Table S2 in Multimedia Appendix 1. These results suggest a correlation between a profile’s richness and popularity. Patients were more interested in physicians with advanced titles, doctoral degrees, social activities, scientific achievements, and other institutional visit experiences.

### Subgroup Analysis

**Figure 4:**
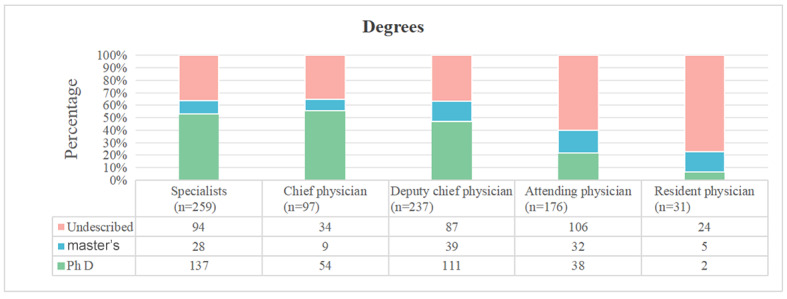
The proportion of degrees in each title group. The results show that the proportion of advantaged items increases with the increase of professional titles.

**Figure 5:**
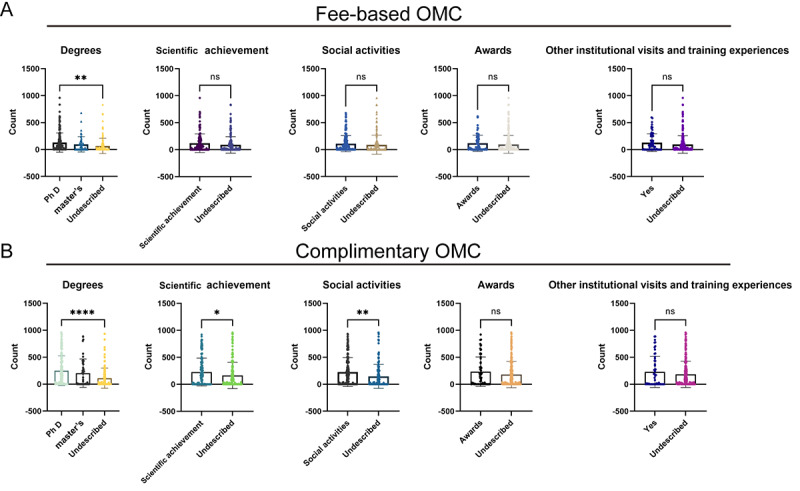
Parts A and B represent the difference analyses of degrees, social activities, scientific achievements, visits to other institutions, and awards factors in the chief physicians and deputy chief physician groups. In the chief physicians and deputy chief physician groups, the maximum value with a large dispersion degree was removed, and 330 people remained. Doctoral degrees remain attractive to patients when data are limited to chief physicians and deputy chief physicians. OMC: online medical consultation. ns: not significant.

Descriptive statistics were used to compare the proportion of degrees, social activities, scientific achievements, visits to other institutions, and awards in each title group. The results showed that the proportion of advantaged items increased with the increase of professional titles (Figure 4 and Figure S2 in Multimedia Appendix 1). In the chief physician and deputy chief physician groups, the maximum value with a large dispersion degree was removed, and 330 people remained. The distribution of complimentary OMC and fee-based OMC visit counts was approximately normal. There was a significant difference in the number of fee-based OMCs between the PhD and the undescribed groups (*P*<.001). There was no significant difference in the number of fee-based OMCs between the master’s degree and undescribed groups (*P*=.22). Social activities, scientific achievements, visits to other institutions, and awards did not affect the number of fee-based OMC visits. There was a significant difference in complimentary OMC visit counts between the PhD and the undescribed groups (*P*<.001). There was a significant difference in consultation volume between the master’s degree and undescribed groups (*P*=.01). Similar results were found for social activities (*P*=.003) and scientific achievement (*P*=.01). There was no difference between the other institutional visit experiences and the undescribed groups (*P*=.35). There was no difference between the awards and the undescribed groups (*P*=.14). The results are shown in Figure 5. These results suggest that richer social activities, scientific achievements, other institutional visit experiences, and awards are more common among people with advanced professional titles. Doctoral degrees remain attractive to patients when the data are limited to chief physicians and deputy chief physicians.

### Data Dimensionality Reduction

**Figure 6:**
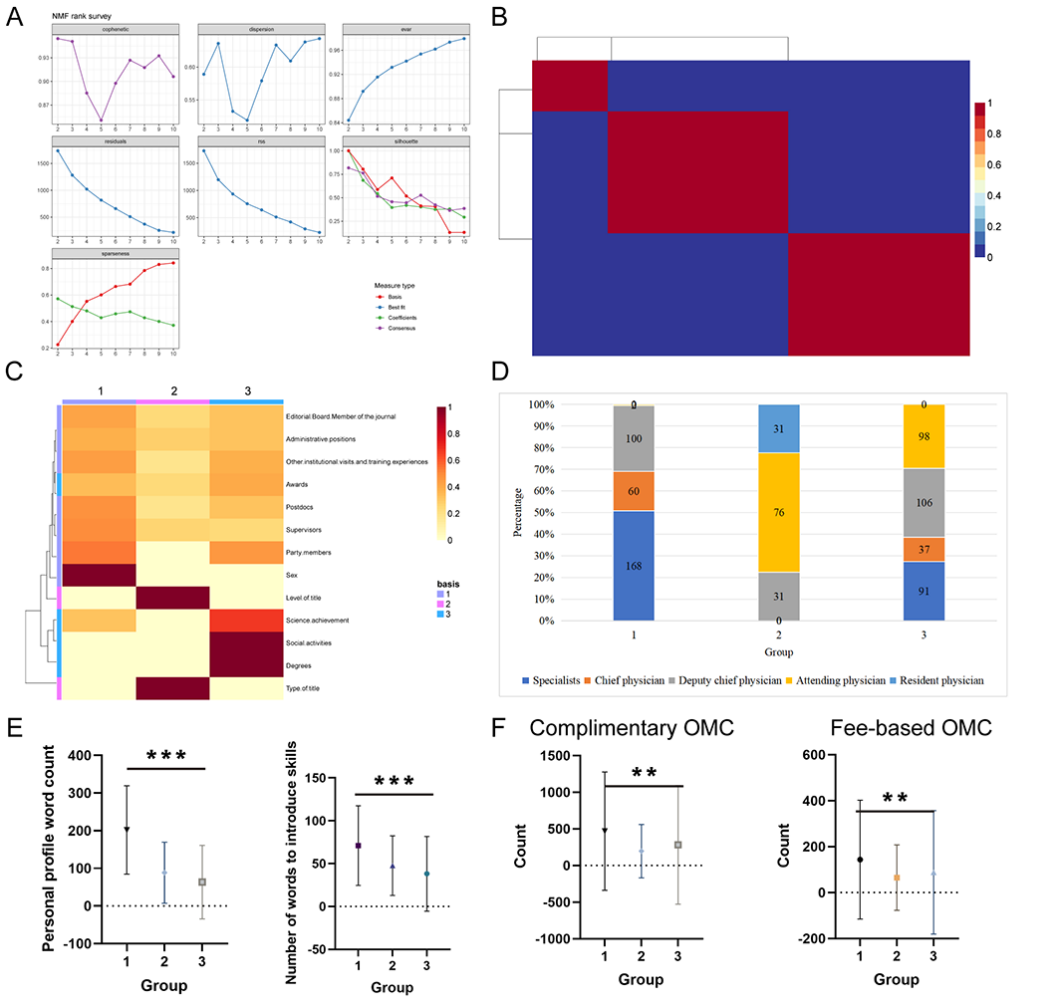
Nonnegative matrix factorization (NMF) data dimensionality reduction. (A) NMF rank survey (k=3 was determined as the optimal cluster number); (B) the data were divided into 3 groups; (C) the heatmap showed significant differences among the 3 groups; (D) the distribution of professional titles in each group was subsequently verified, indicating that the first group had significantly more senior professional titles than the other two groups; (E) analysis of the difference in the number of words in personal introduction and skill introduction between groups. (F) analysis of the difference in consultation volume between groups. Complimentary online medical consultation (OMC; *P*=.004) and fee-based OMC (*P*=.007) visit counts showed a significant difference among the 3 groups. * represents *P*<.05, ** represents *P*<.01, and *** represents *P*<.001.

In this analysis, NMF was used as a dimensionality reduction technique to calculate the parental correlation coefficient through the NMF rank survey, and k=3 was determined as the optimal cluster number (Figure 6A). Therefore, the data were divided into 3 groups, with 330 people in the first group, 138 people in the second group, and 332 people in the third group (Figure 6B). The 13 primary vectors in the NMF matrix showed significant differences among the 3 groups (Figure 6C), and the distribution of professional titles in each group was subsequently verified, indicating that the first group had significantly more senior professional titles than the other two groups (Figure 6D). The results showed that the richness of personal introduction among the groups was different, with mean numbers of 201.8, 88.37, and 63.42, respectively (*P*<.001). The mean number of skill introduction words for the 3 groups was 71.05, 47.69, and 38.17, respectively (*P*<.001); the results are shown in Figure 6E. Complimentary OMC (*P*=.004) and fee-based OMC visit counts (*P*=.007) showed a significant difference among the 3 groups (Figure 6F). These results suggest that medical staff with advanced titles, social activities, scientific achievement, and doctoral degrees are more trusted by patients.

## Discussion

### Principal Findings

Internet hospitals are emerging in China as a new way for physicians to use telecommunications technology for health care services and education [24]. The characteristics of patient departments and diseases in internet hospitals are consistent with the characteristics of superior disciplines in entity hospitals [25]. In this study, we first constructed a static information data pool for doctors, with data sorted by professional title level, professional title type, party membership, degree, postdoctoral experience, supervisory status, administrative position, social activities, scientific achievements, journal editorial board membership, visits to other institutions, awards, and gender. Correlation analysis results indicated a relationship between the richness of personal information and popularity. Patients tend to be more interested in doctors with senior professional titles, doctoral degrees, involvement in social activities, scientific achievements, and experience visiting other institutions. When the data are limited to chief physicians and deputy chief physicians, a doctoral degree remains attractive to patients. Subsequent dimensionality reduction of the data suggests that medical professionals with senior titles, involvement in social activities, scientific achievements, and doctoral degrees are more trusted by patients.

The First Affiliated Hospital of Guangxi Medical University is located in the economically underdeveloped areas of western China. In September 2020, it was the first public hospital to obtain an internet hospital license [26,27] and ranked 41 in China’s comprehensive strength in 2022 [28]. In 2023, a total of 85.25% of the in-person outpatient services of the First Affiliated Hospital of Guangxi Medical University came from internet hospital appointment registrations. Hospital physicians in public hospitals are busy and seldom take the initiative to manage their personal brands, and they also lack the knowledge and ability to promote themselves effectively. In 2023, only 74 physicians had more than 1000 consultations, while most had fewer than 200 visits. The data exhibit a star effect, and the results may be related to platform recommendation rules. Wang et al’s [29] research suggests that compared to township hospitals, doctors in tertiary hospitals are less willing to provide services in internet hospitals. Some studies suggest that patients are more willing to consult physicians with a higher web-based workload [30]. When strongly recommended by physicians, patients are more likely to choose remote consultations [31]. Our results suggest that there are differences in the data distribution of complimentary OMCs and fee-based OMCs, with a strong correlation. Subsequent results also confirmed that when adopting a no-cost counseling strategy, patients seemed to care about the doctor’s degree, social activities, scientific achievement, other institutional visits, and awards. When patients had to pay the cost to purchase web-based medical services, they became more careful and considered factors related to professional titles. However, data based on the Good Doctor Platform argues that higher titles and more complex profiles reduce potential patients’ initial trust in physicians and limit patient choices [13]. Higher professional titles require longer work experience, leading to more advantages. Subgroup analysis results suggest doctoral degrees remain attractive to patients when data are limited to chief physicians and deputy chief physicians. The results of data dimensionality reduction suggest that medical personnel with advanced titles, social activities, scientific achievements, and doctoral degrees are more trusted by patients. Web-based doctor information not only affects patients’ choice of remote medical treatment but also determines whether patients switch from physicians’ remote medical services to in-person medical services [32], which is also one of the differences between the two modes of establishing internet hospitals in public hospitals and hosting third-party institutions. Our study found no difference in the number of fee-based OMCs between specialists and residents. The reason may be financial problems. Doctors with higher outpatient titles, better digital reputations, and higher past sales tend to charge higher consultation fees [33]. In low-income African regions, patients rated physicians’ trustworthiness only through interpersonal behavior and technical skills [34]. The diversity of patients leads to differences in their ability to identify information about physicians [35]. Therefore, more factors (ie, patients’ symptoms, diagnoses, and geographic locations) should be included in digital physician referral models [36]. When exploring how internet hospitals influence patient choice behavior, previous research conducted in traditional hospital settings provides valuable insights. Roh et al’s [37] study, although focused on determinants of patient choice in rural hospitals, offers an enlightening analysis of service scope and market competition, informing the expansion of service and competitive strategies in internet hospitals. Furthermore, Sivey [38] examined the significance of waiting time and distance in patient choice, highlighting the inherent advantages of internet hospitals in providing convenient services and reducing wait times. In comparing patient choice patterns across different health care settings, Ruwaard and Douven’s [39] study revealed an interesting phenomenon: patients with cataracts tended to choose the most popular institutions when selecting physical hospitals, indicating that digital platform reputation and word of mouth were equally important for internet hospitals. Guarducci et al [40] emphasized the profound impact of hospital resource allocation on patient mobility, stressing the crucial role of optimizing resources, including physician workforce, technical support, and patient management systems, in enhancing service efficiency and patient satisfaction for internet hospitals [40]. Therefore, it is imperative for internet hospitals to prioritize patient feedback and service quality improvement to establish a favorable digital reputation. By allocating resources effectively, internet hospitals can reduce operational costs and respond more precisely to patient needs, and this enhances their attractiveness and competitiveness.

### Limitations

Powell and Deetjen [41] used a mixed methods approach to identify 6 distinct types of health internet users, reflecting the varying orientations people have when using the internet to seek health information. The study found through investigation and analysis that only 37.1% of patients with cancer at Parkland Memorial Hospital had access to the internet, and those who used the internet were more likely to be younger and have a higher level of education [42]. We did not categorize patients because of the data structure. For example, for patients who are critically ill or in urgent need of treatment (eg, from the organ transplantation department, emergency department, and intensive medicine department), fee-based OMCs in internet hospitals seem to be of little help, and the data of these patients and professional physicians may be considered excluded. However, as some patients may switch from remote consultations to in-person visits, these doctors are also factors to be considered when patients choose where to seek medical treatment [43].

### Conclusions

As an OMC service of public hospitals, internet hospitals serve as a platform for hospital publicity and physicians’ displays. Physicians’ “personal advertising” on the platform is an important medium for gaining patients’ trust. The results of this study can help physicians better present themselves on digital platforms to increase their digital visits. It also helps hospital managers formulate and improve internet hospitals’ relevant quality control standards and regulations so that both physicians and patients can enhance their medical behaviors. In the future, we will start from the hospital perspective, subdivide patients according to customer characteristics, and recommend personalized doctor information display according to patient groups.

## Appendix

Multimedia Appendix 1 Additional statistics.

## Abbreviations

NMF: nonnegative matrix factorization
OMC: online medical consultation

## Data Availability

The data used in this research are available upon individual request from the corresponding author.
