# Supplementary material for: The Relationship Between Static Characteristics of Physicians and Patient Consultation Volume in Internet Hospitals: Quantitative Analysis
Source: JMIR Form Res. 2024 Jun 17;8:e56687. doi: 10.2196/56687 (PMC11217713; doi:10.2196/56687)
Supplement: Multimedia Appendix 1 [file formative_v8i1e56687_app1.pdf]

The Relationship between Static Characteristics of Physicians and Patient Consultation Volume in Internet Hospitals: A Quantitative Analysis

Abstract

Background: Internet medical treatment, also known as telemedicine, represents a paradigm shift in healthcare delivery. This contactless model allows patients to seek medical advice remotely, often before they physically visit a doctor's clinic. Herein, physicians are in a relatively passive position as patients browse and choose their healthcare providers. While a wealth of experience is undoubtedly a draw for many patients, it remains unclear which specific facets of a doctor's credentials and accomplishments patients prioritize during their selection process.

Objective: Our primary aim is to delve deeper into the correlation between physicians' static characteristics—such as their qualifications, experiences, and online profiles—and the number of patient visits they receive. We seek to achieve this by analyzing comprehensive internet hospital data from public hospitals. Furthermore, we aim to offer insights into how doctors can present themselves more effectively on online platforms, thereby attracting more patients and improving overall patient satisfaction.

Methods: We retrospectively gathered online diagnosis and treatment data from the First Affiliated Hospital of Guangxi Medical University in 2023. This data underwent rigorous analysis, encompassing basic descriptive statistics, correlation analyses between key factors in doctors' online introductions, and the number of patient consultation visits. Additionally, we conducted subgroup analyses to ascertain the independence of these vital factors. To further distill the essence from this data, we employed Non-negative Matrix Factorization to identify crucial demographic characteristics that significantly impact patient choice.

Results: The statistical results suggested that there were significant differences in the distribution of consultation volume and consultation volume ($P<.01$), and the correlation analysis results suggested that there was a strong correlation between the two groups of data ($r_s=0.93$, $P<.01$). There is a correlation between the richness of a profile and popularity ($P<.01$). Patients were more interested in physicians with advanced titles, doctorates, social activities, scientific achievements, and Other institutional visit experiences($P<.05$). More prosperous social activities, scientific achievements, experiences of other institutional visits, and awards are more common among people with advanced professional titles than others. Doctoral degrees remain attractive to patients when the data is limited to senior physicians($P<.01$). Patients trust the medical staff with

advanced titles, social activities, scientific achievements, and doctoral degrees(P<.01).

Conclusions: Patient preferences while choosing a healthcare provider differ notably when comparing free versus paid consultation options. Notably, patients tend to place greater trust in doctors who have achieved advanced professional titles and are more likely to seek out those with doctoral qualifications compared to other professional ranks. Additionally, physicians who actively participate in social events and scientific endeavors often find themselves at an advantage when attracting new patients. Given these insights, doctors who invest in enhancing their personal and professional experiences within these domains are likely to experience increased popularity and patient satisfaction.

Keywords: Static characteristics of physicians; Internet hospitals; Telemedicine; statistical analysis; Online consultation

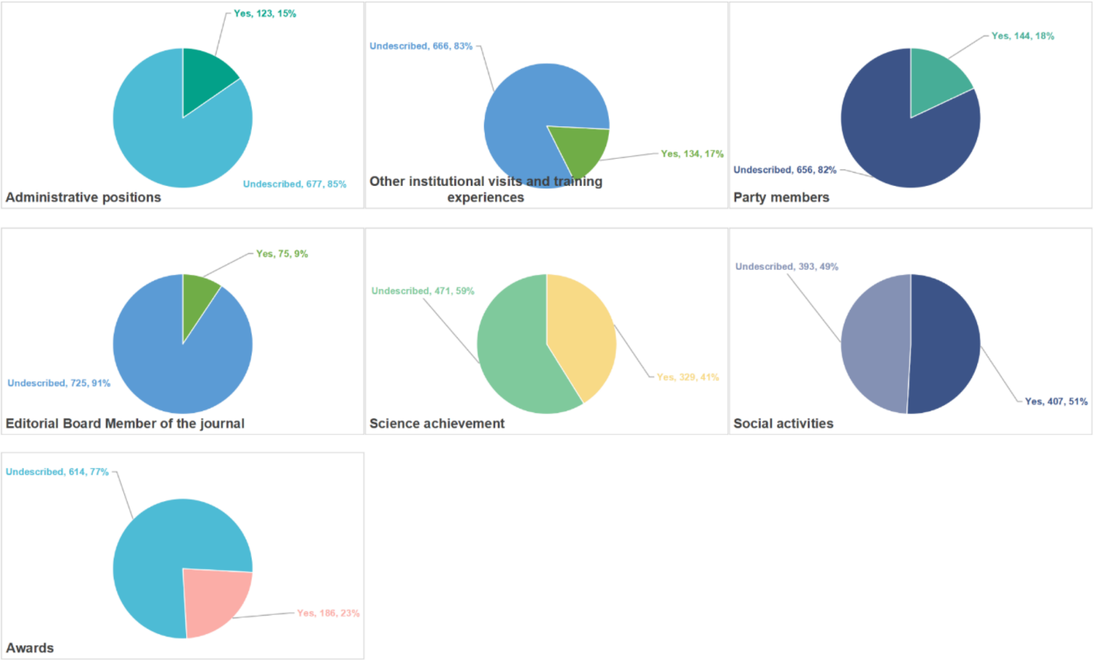

Fig.S1 Distribution ratio results of internet hospital doctor static information data.

Table S1. The relationship between personal information word count data and consultation volume.

Group: A	Group: B	Wilcoxon	rs (Spearman)	P value
Personal profile word count	Number of fee-based OMC	<0.01	0.26	<0.01
	Number ofcomplimentary OMC	<0.01	0.24	<0.01
Number of words to introduce skills	Number of fee-based OMC	0.85	0.36	<0.01
	Number of complimentary OMC	<0.01	0.33	<0.01

Table S2. Relationship between 13 physician static characteristics and OMC volume.

Group: A		Group: B	fee-based OMC Median(A)	fee-based OMC Median(B)	P value	complimentary OMC Median(A)	complimentary OMC Median(B)	P value
Male	vs.	Female	5.00	8.00	0.79	28.00	36.00	0.74
Junior professional titles	vs.	Senior professional titles	23.00	5.00	0.02	85.00	16.00	0.19
	vs.	Intermediate professional titles	0.00		0.43	0.00		0.10
Resident physician	vs.	Specialists	0.00	5.00	0.17	16.00	1.00	0.50
	vs.	Chief physician	33.00		0.01		83.00	0.14
	vs.	Deputy chief physician	44.00		0.01		126.00	0.08
	vs.	Attending physician	0.00		0.43		0.00	0.10
Party members	vs.	Undescribed	13.50	5.00	0.20	29.00	29.00	0.39
Ph.D.	vs.	Undescribed	46.00	0.00	<0.01	155.50	0.00	<0.01
Master	vs.		4.00		0.06	23.00		0.08
Postdocs	vs.	Undescribed	49.00	5.00	0.08	342.00	28.00	0.07
Supervisors	vs.	Undescribed	0.00	6.50	0.43	0.00	32.00	0.35
Administrative positions	vs.	Undescribed	14.00	5.00	0.33	27.00	29.00	0.65
Social activities	vs.	Undescribed	33.00	0.00	<0.01	116.00	2.00	<0.01
Science achievement	vs.	Undescribed	33.00	1.00	<0.01	108.00	11.00	<0.01
Editorial Board Member of the journal	vs.	Undescribed	17.00	5.00	0.12	29.00	29.00	0.14
Other institutional visits and training experiences	vs.	Undescribed	58.50	3.00	<0.01	252.50	14.50	<0.01
Awards	vs.	Undescribed	30.50	3.50	<0.01	101.50	22.50	0.04

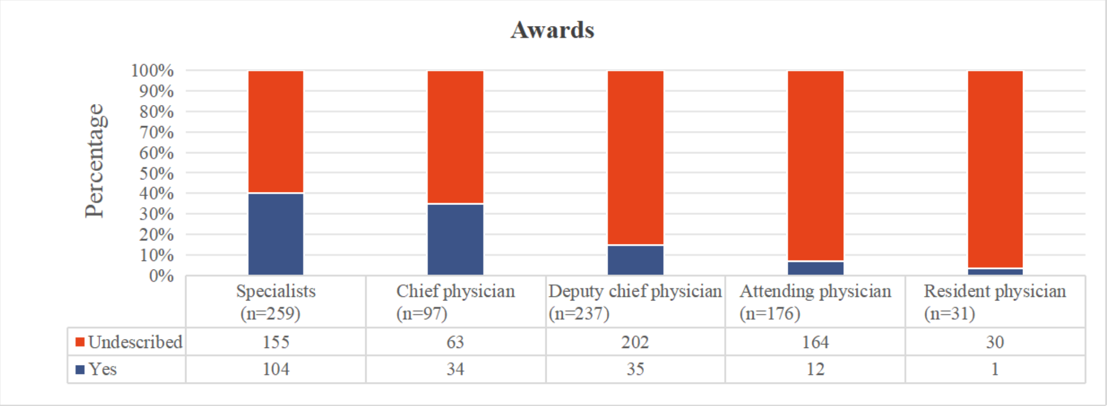

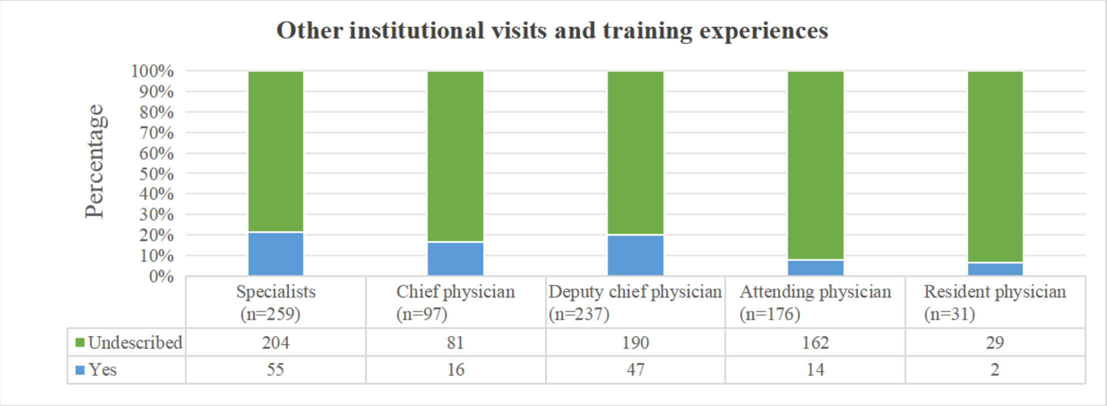

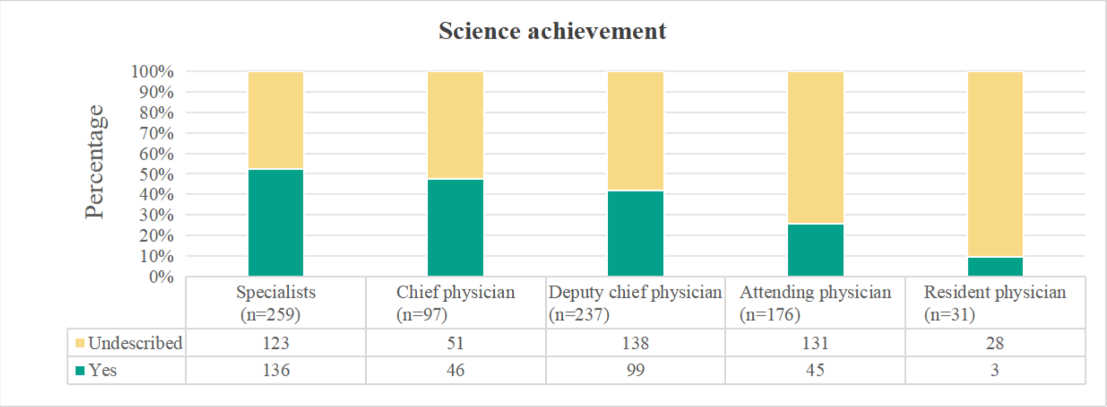

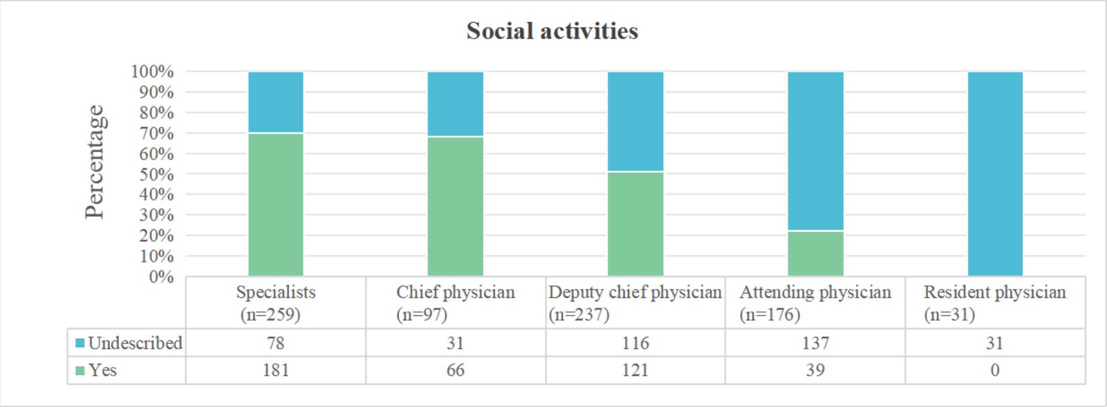

Fig.S2 The proportion of social activities, scientific achievements, visits to other institutions, and awards in each title group.
